# Willingness of a Multiethnic Immigrant Population to Donate Biospecimens for Research Purposes

**DOI:** 10.1007/s10903-021-01241-4

**Published:** 2021-07-26

**Authors:** Amy Raines-Milenkov, Martha Felini, Eva Baker, Rushil Acharya, Elvis Longanga Diese, Sara Onsa, Hlawn Iang, Anab Abdi, Idara Akpan, Arbaz Hussain, Teresa Wagner, Jonathan Hughes

**Affiliations:** Department of Pediatrics and Women’s Health, University of North Texas Health Science Center, Texas College of Osteopathic Medicine, Fort Worth, TX USA

## Abstract

This cross-sectional study explores the willingness to donate biospecimens for research purposes among six refugee communities in North Texas (spanning Myanmar, Central Africa, Somalia, Nepal, Arabic speaking countries, and others). Participants were asked four questions about biospecimen donation: (1) previously asked to donate, (2) ever agreed to donate, (3) willingness to donate for future research, and (4) what samples they would be willing to donate. Most participants (77%) were willing to donate biosamples for medical research; 58% were willing to donate samples. Fewer refugees from Somalia were willing to donate compared to immigrants from Myanmar, Central Africa, and Nepal (*p* < 0.01). Participants in the older age group (40 + years) were 3.2 times more likely to be willing for donation of biospecimens than the younger ones (OR 3.22, 95% CI 1.22, 8.55). Findings suggest refugees’ willingness to participate in biospecimen donation which support intentional inclusion of multicultural populations into medical research.

## Introduction/Background

Immigrants face disparities in early cancer identification, treatment, and death compared to the general United States-born population [1–4]. Lower uptake of preventive cancer screenings may account for some of this disparity [5–7]. Not only are immigrants less likely to receive preventive screenings, but they may also be less likely to participate in clinical trials and basic research focused on cancer prevention and treatment in minority populations. Most cancer prevention research with immigrants are focused on the behavioral, health care system, and sociocultural environment domains within the National Institute on Minority Health Disparities Research Framework, with less attention to biological vulnerability and mechanisms [8].

Exploring demographics, social determinants, health care utilization, and outcomes, while incorporating biomedical research, however, has the potential to accelerate our understanding of disease risks and processes leading to disease prevention, early detection, and improved treatment and survival [9]. Diversity in these demographic and biological parameters is essential to ensure the generalizability and validity of new findings and treatment options [10]. Biospecimens, including tissues, blood, and urine are usually, but not always obtained during diagnostic tests or procedures. These samples can be used in risk stratification of patients, research, and identification of new causal relationships and treatments, and in precision care for that patient. Precision or personalized cancer care takes individual variability into account in prevention and treatment based on the genomic and proteomic profile of the cancer cells. Examples of precision medicine in cancer care include the use of Trastuzumab in Her-2 positive breast cancer and the use of Imatinib in BCR/ABL positive chronic myeloid leukemia [11, 12]. These advances are exciting, but are dependent on the donation of biospecimens to conduct the necessary research.

Minorities, including immigrants, however, are underrepresented in cancer research involving biospecimens [10, 13, 14]. This lack of representation is unfortunate because it limits the benefits of biospecimen collection to better understand humans in general, and for underserved populations in particular. There is a general agreement, supported by science and social justice needs, about the importance of having a diverse population participating in biomedical research [14]. Disproportionately affected populations must be included in research studies to help reduce persistent and troubling health disparities. Evidence suggests that not having enough blood donors from ethnic minority groups negatively affects the number of available biospecimens, and also limits the ability to address specific health issues in these populations [13].

There are few studies exploring the willingness to participate in biospecimen donation for research purposes among racial/ethnic minorities, and most of the studies are limited to African American and Latino communities. Research from these similarly marginalized communities suggest that minority groups are generally willing to participate in biospecimen research but have barriers to participation [10, 13]. Some of these barriers include medical mistrust, a perceived lack of benefits, and fear of physical harm after donating biospecimen samples [15].

Ethnicity, age, level of education, and area of residence have been found as predictors for biospecimen donation. Being African American or Hispanic, not having a high school diploma, or being aged between 35 and 44‐year‐olds have been associated with lower odds of donating biospecimen samples [13]. On the other hand, education on biospecimen collection and awareness of its benefit for future generations has been associated with increased willingness to donate among minority groups [10].

Despite the presence of diverse ethnic groups in the U.S., including immigrants, and the potential of discoveries with their contribution, little is known about how to engage immigrant populations or their willingness to submit biospecimens for medical research. The purpose of this study was to examine the willingness to donate biospecimens for research purposes among a diverse multi-ethnic population of immigrants. Findings from this study can be used to develop strategies to engage multiethnic populations in studies that include biospecimen donation.

## Materials and Methods

Trained, bilingual and bicultural community health workers (CHW) recruited participants from six immigrant communities representing 24 different countries, in a cancer prevention and research project called the Building Bridges Initiative (BBI). The BBI is a community outreach intervention of the University of North Texas Health Science Center Department of Pediatrics and Women’s Health funded by the Cancer Prevention and Research Institute of Texas (CPRIT). In 2014, we were awarded a 3-year prevention program grant to fund the development and implementation of evidence-based education and navigation services to breast and cervical cancer screening, and Hepatitis B screening among refugees in the North Texas area. The intervention was expanded to include colon cancer screening, HPV vaccination for adolescents in the second three-year funding cycle. Components of this community health worker model include: Community Leader Engagement, Culturally Adapted Education, Navigation and Enabling Services to link to Trauma-Informed Screenings, Testing and Vaccination. During enrollment participants were asked demographic, migration history, cancer awareness and history, previous cancer screenings, vaccination receipt, and questions about willingness to donate biospecimen, health behaviors among others. Participants were consented in their native language either verbally, written format, or both methods, depending upon the participant’s literacy.

Four questions about biospecimen donation included: (1) Have you been asked to give a small sample of blood, saliva, tissue or other biological sample for medical research? Answer options: ‘Yes’, ‘No’, ‘I don’t know’; If ‘Yes’: ‘Did you agree to donate the sample?’ ‘Yes,’ ‘No,’ ‘I don’t know’; (2) ‘Some biological samples can be collected by you in the privacy of your own home (urine or saliva). Would you be willing to participate in this type of research by collecting a sample at home?’ Answer options: ‘Yes,’ ‘No’, ‘I don’t know’; (3) ‘Other biological samples need to be collected by a doctor or trained health professional (e.g. Blood). Would you be willing to give a biological sample for research in a clinic?’ (4) ‘In the future, would you be willing to give the following biological specimen for research purposes in a study?’ Answer options: Blood, Saliva, Urine, Stool Sample, Hair, Nail Clippings. Participants were not compensated for their involvement in the study or intervention. The North Texas Regional Institutional Review Board approved this study.

After institutional review board approval and informed consent were obtained, a total of 171 Building Bridges program participants enrolled between August 2017 and November 2018 were asked biospecimen donation questions. Participants self-reported their country of origin and primary language along with other baseline demographic characteristics. For the purpose of this cross-sectional analysis, participants were categorized into six major immigrant groups based on their country of origin, cultural similarity, and primary language: (1) Myanmar group (including participants from Myanmar and Thailand), (2) Central African Region (including participants from Congo, Rwanda, Burundi, and Kenya who came from the Great Lakes Region with a similar history and languages), (3) Somalia group (including participants from Somalia only), (4) Nepal group (including participants from Nepal and Bhutan), (5) Arabic Speaking Countries (including participant from Sudan only), and, (6) Others group (includes participants from Chad and Eritrea).

Descriptive statistics of baseline demographic characteristics (Table 1) and proportions for willingness to donate biospecimen (Table 2) were calculated across cultural groups. Appropriate tests of independence such as Pearson’s Chi-square test and Fisher’s Exact test were performed to determine if there is any association between willingness to donate and each of the potential confounders/covariates. All descriptive and statistical analyses were carried out using SAS® 9.4 software and p-values less than 0.05 were considered statistically significant.

**Table 1:** Baseline demographic characteristics of building bridges program participants who were asked for their willingness on bio-specimen donation by Cultural Group, Fort Worth, Texas, August 2017–November 2018 (*N* = 171)

Characteristic	Total	Myanmar^a^	Central African Region^b^	Somalia	Nepal^c^	Arabic speaking Countries^d^	Others^e^	Test of independence
# of Participants,* n* (%)	171 (100)	89 (52)	36 (21)	22 (13)	15 (9)	7 (4)	2 (1)	
*Gender*	171 (100)							Fisher’s exact
Female	152 (89)	80 (90)	31 (86)	21 (95)	12 (80)	6 (4)	2 (100)	*p*-value = 0.04
Male	19 (11)	9 (10)	5 (14)	1 (5)	3 (20)	1 (5)	0 (0)	
*Age* (in years),* n* (%)	171 (100)							Pearson’s χ2
18–29	46 (27)	29 (33)	7 (19)	9 (41)	1 (7)	0 (0)	0 (0)	*p*-value = 0.02
30–39	60 (35)	36 (40)	13 (36)	5 (23)	2 (13)	2 (29)	2 (100)	
40–49	31 (18)	11 (12)	7 (19)	6 (27)	3 (20)	4 (57)	0 (0)	
50–59	17 (10)	7 (8)	5 (14)	2 (9)	2 (13)	1 (14)	0 (0)	
60 or above	17 (10)	6 (7)	4 (11)	0 (0)	7 (47)	0 (0)	0 (0)	
*Marital status*,* n* (%)^*^	151 (88)							Pearson’s χ2
Married	110 (73)	66 (82)	11 (37)	15 (71)	12 (100)	5 (83)	1 (50)	*p*-value = 0.31
Other^**^	41 (27)	14 (18)	19 (63)	6 (29)	0 (0)	1 (17)	1 (50)	
*Current health insurance*,* n* (%)^*^	169 (99)							Pearson’s χ2 *p*-value = 0.30
Yes	42 (25)	19 (22)	12 (33)	4 (18)	3 (20)	3 (43)	1 (50)	
No	127 (75)	68 (78)	24 (67)	18 (82)	12 (80)	4 (57)	1 (50)	
*Formal education*,* n* (%)^*^	121 (71)							Pearson’s χ2
Less than 12 years	76 (63)	44 (77)	17 (57)	14 (78)	1 (14)	0 (0)	0 (0)	*p*-value = 0.23
12 years or more	45 (37)	13 (23)	13 (43)	4 (22)	6 (86)	7 (100)	2 (100)	
*How well do you speak English?*,* n* (%)^*^	170 (99)							Pearson’s χ2 *p*-value = 0.73
Not at all/not well	121 (71)	69 (78)	25 (69)	14 (67)	11 (72)	2 (29)	0 (0)	
Well/very well	49 (29)	20 (22)	11 (31)	7 (33)	4 (27)	5 (71)	2 (100)	
*Years lived in US*,* n* (%)	171 (100)							Pearson’s χ2
Less than 5 years	100 (58)	38 (43)	30 (83)	17 (77)	8 (53)	6 (86)	1 (50)	*p*-value = 0.87
5–10 years	59 (35)	46 (52)	4 (11)	4 (18)	4 (27)	0 (0)	1 (50)	
More than 10 years	12 (7)	5 (5)	2 (6)	1 (5)	3 (20)	1 (14)	0 (0)	

^a^Includes participants from both Myanmar (*n* = 86) and Thailand (*n* = 3)

^b^Includes participants from: Congo (*n* = 26), Rwanda (*n* = 7), Burundi (*n* = 2), and Kenya (*n* = 1, who reported Swahili as native language)

^c^Includes participants from both Nepal (*n* = 11) and Bhutan (*n* = 4)

^d^Includes participants from Arabic speaking countries: Sudan (*n* = 7)

^e^Includes participants from: Chad (*n* = 1) and Eritrea (*n* = 1)

^f^*p*-values resulted from the test of independence from ‘willingness to donate biospecimen’

^*^Owing to missing data, numbers may not total to 171

^**^Includes Single, Divorced, Separated, Widowed, Cohabitating, or Declined to answer

**Table 2:** Willingness for bio-specimen donation among building bridges program participants by Cultural Group, Fort Worth, Texas, August 2017–November 2018 (*N* = 171)

Characteristic	Total	Myanmar^a^	Central African Region^b^	Somalia	Nepal^c^	Arabic speaking Countries^d^	Others^e^
*Previously asked to donate?*	171 (100)	89 (52)	36 (21)	22 (13)	15 (9)	7 (4)	2 (1)
Yes	48 (28)	35 (39)	5 (14)	0 (0)	7 (47)	1 (14)	0 (0)
No/I don’t know	123 (72)	54 (61)	31 (86)	22 (100)	8 (53)	6 (86)	2 (100)
*If previously asked to donate, ever agreed to donate? n* (%)	47 (98)						
Yes	46 (98)	34 (97)	5 (100)	0 (0)	6 (100)	1 (100)	0 (0)
No/I don’t know	1 (2)	1 (3)	0 (0)	0 (0)	0 (0)	0 (0)	0 (0)
*Willing to donate biological specimen in future?*	168^*^ (98)						
Yes	130 (77)	62 (70)	34 (94)	11 (55)	15 (100)	6 (86)	2 (100)
No/Don’t know	38 (23)	26 (30)	2 (6)	9 (45)	0 (0)	1 (14)	0 (0)
Wald χ^2^ *p*-value	0.02^**^	–	0.02^**^	0.07	0.11	0.81	0.68
Odds Ratio (95% CI)		–	5.71 (1.38, 23.75)	0.38 (0.13, 1.09)	13.65 (0.58, 322.91)	1.30 (0.16, 10.53)	2.23 (0.05, 95.21)

*CI* Confidence interval

^a^Includes participants from both Myanmar (*n* = 86) and Thailand (*n* = 3)

^b^Includes participants from: Congo (*n* = 26), Rwanda (*n* = 7), Burundi (*n* = 2), and Kenya (*n* = 1, who reported Swahili as native language)

^c^Includes participants from both Nepal (*n* = 11) and Bhutan (*n* = 4)

^d^Includes participants from Arabic speaking countries: Sudan (*n* = 7)

^e^Includes participants from: Chad (*n* = 1) and Eritrea (*n* = 1)

^*^Owing to missing data, numbers may not total to 171

^**^P < 0.05

## Results

Table 1 includes descriptive statistics of baseline characteristics of all 171 study participants, stratified by cultural group. Almost two-third (63%) of women were under 40 years of age at enrollment, whereas a little over half (53%) of men were 40 or older. Overall, 61% of all participants were below 40 years of age (Mean = 38, Median = 34, SD = 14). With the exception of the Central African Region, at least 50% from each group reported to be married. More men (71%) reported never attending any school or receiving less than 12 years of formal education than women (61%). Over half, 58%, stated that they have been in the US for less than 5 years and more than two-thirds (71%) of participants reported not speaking English well or not at all. 63% reported never receiving any formal education or attending less than 12 school-years as their highest level of education. The majority of participants, 75%, did not have health insurance coverage at the time of enrollment into the BBI program. *P*-values resulting from the test of independence are declared in Table 1. Age (*p* = 0.02) and gender (*p* = 0.04) were found to be predictors of willingness to donate.

In Table 2, we present willingness of BBI participants to donate biospecimens for medical research in the past, as well as in the future, and compare willingness across six immigrant/refugee communities. Most (77%) were willing to donate bio samples for medical research; 58% were willing to donate all 6 types of specimens, including blood, saliva, urine, stool, hair, and nail clippings. More Females (80%) were willing to donate than men (50%). More than a quarter, 28% of all participants, self-reported that they had been asked to give a small sample of blood, saliva, tissue or other biological sample for medical research in the past.

**Fig. 1:**
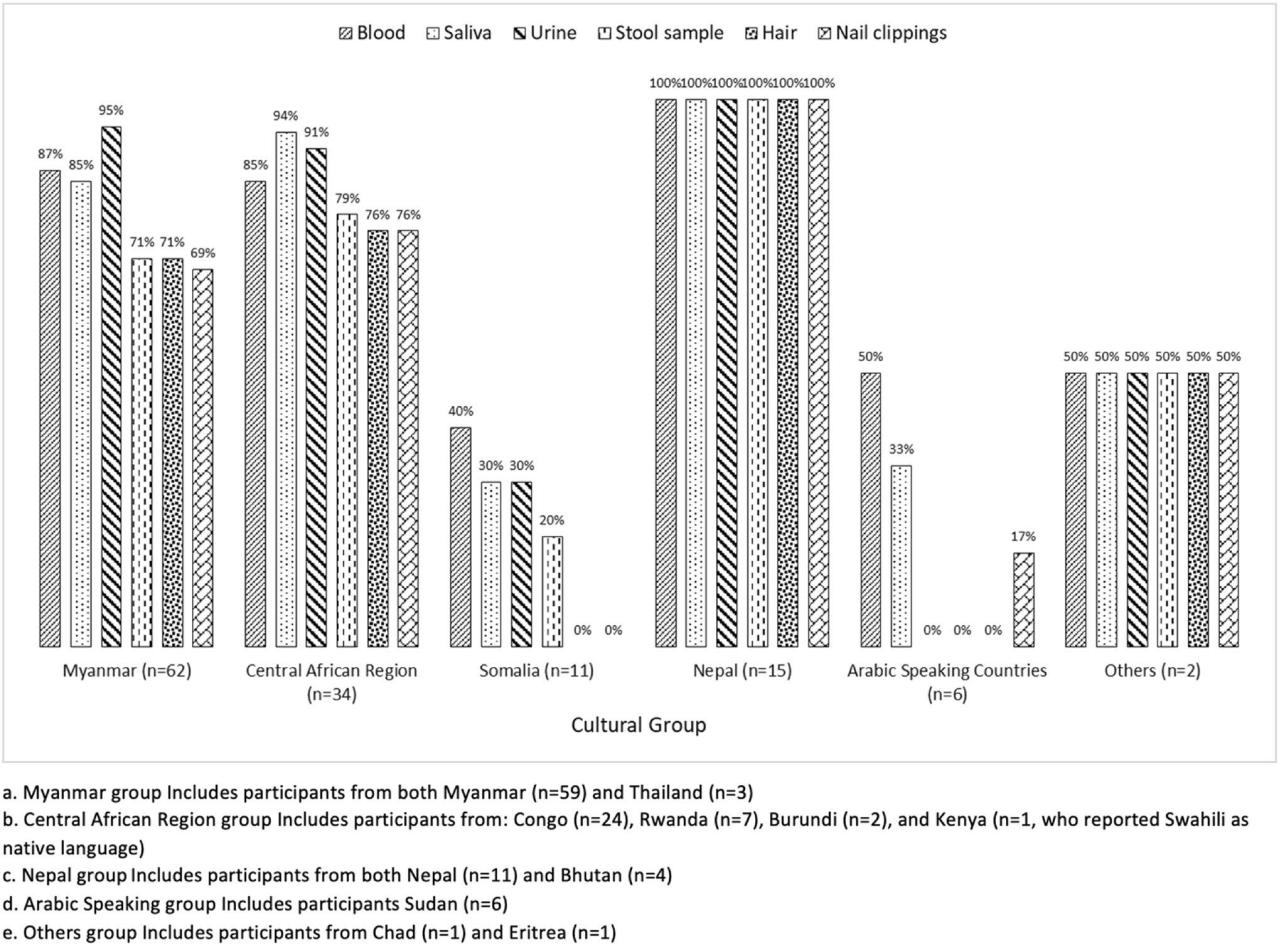
Types of biospecimens building bridges programs participants willing to donate for research purposes by Cultural Group, Fort Worth, Texas, August 2017–November 2018 (*N* = 130)

Overall, fewer Somalis (55%) were willing to donate in the future if asked; compared to Myanmar (70%), Central Africa (94%) and Nepal (100%). Differences in willingness to donate across six groups were examined using Firth’s Penalized Likelihood regression method. After controlling for age and gender, prevalence odds ratios (POR) and 95% confidence intervals (CIs) were calculated for each cultural group to assess the association between willingness to donate (dependent variable: yes/no) and cultural group (independent variable: using Myanmar/Thailand, the largest among all six groups, as the comparison group). Statistically significant association between culture and willingness to donate was observed (*p* = 0.02). The odds of participants from the Central African Region willing to donate biospecimens in the future for research purposes were 5.7 times greater than participants from a Myanmar group (OR 5.71, 95% CI 1.38, 23.75). Participants in the older age group (40 + years) were 3.2 times more likely to be willing for donation of biospecimens than the younger ones (OR 3.22, 95% CI 1.22, 8.55). The odds of women willing to donate in future were approximately 6.2 times more than men (OR 6.19, 95% CI 1.80, 21.34). Figure 1 demonstrates types of biospecimens that participants were willing to donate by cultural group.

## Discussion

Our findings indicate a higher willingness to participate in biospecimen donation than what has been observed in other studies of immigrant and minority populations. In one study, 67% of foreign-born Latinos were willing to participate in biospecimen donation following an education program [16]. In another study, only 46% of Hispanics and 34% of African Americans endorsed their support for blood sample donations prior to educational intervention. These numbers increased respectively to 56% and 36% after education was provided [17].

There may be numerous reasons for this higher than expected reported willingness to donate biospecimens for research purposes. The questions were asked as part of a larger cancer education and screening intervention focused primarily on refugee populations. Hence, participants in this study represent a group that at baseline may be more likely to participate because of their participation in the larger intervention study. In addition, refugee and immigrant community leaders were consulted and engaged during the grant application, intervention design, hiring and on-going monitoring of the program. This may have increased the level of trust between the researchers and participants since many refugees live in tight-knit communities. Recruitment, enrollment and questioning by bicultural and bilingual team members may have also added an element of trust and willingness to participate in donation.

Reported willingness, however, may not translate into action. Previous studies have identified numerous barriers to donation by race/ethnic group including medical mistrust (African American), lack of personal benefit, apprehension about the donation process, usage for research and suspicion of corporate exploitation (White) [15]. Additional research, particularly qualitative research, is needed to identify participation facilitators and inhibitors among diverse immigrant populations. Other studies with immigrant and minority populations have included an education component [17]. This education component may elicit additional questions and concerns about biospecimen donation that we did not identify in our study. With a more in-depth and target education program we may also uncover similar barriers. The discomforts of blood and tissue donations outside of diagnostic procedures may hinder participation. Additionally, the barriers that contribute to poor health outcomes in general for this population, i.e., language, transportation, inconvenient locations and collection times, may also prove to be a barrier for biospecimen donation. The cost of translation and interpretation to assure a full consent and understanding may be a barrier for researchers.

Our study found that only age and gender predicted willingness to donate biospecimens with females and individuals 40 and older more likely to participate. Older individuals have been found to be more willing to donate biospecimens in both immigrant, as well as the US general population [10, 13, 18]. In another study involving a large sample of African women, the odds of participating in biospecimen donation significantly increased with age, from 48.6% among women aged below 40 to 63.1% among those 60 years and older [18]. Older individuals’ willingness to donate biospecimens may be explained by the hope that it will benefit future generations [10].

The gender difference observed in our study was not consistent with findings from other studies which either suggested that there were no gender difference in the intent for biospecimen donation [19] or men being more likely to donate biospecimen than women [20, 21]. The difference in findings from other studies could be the result of our study including only immigrant populations with most of them coming to the US as refugees. Hence, the greater intent to donate biospecimens observed among women in this study could be explained by the high pregnancy rate among refugee women that make them more used to medical procedures and tests [22]. In addition, a study involving US underserved communities found that women were 69% more likely to have knowledge on biospecimens compared to men [23].

Potential threats to internal validity that could have resulted from social desirability bias were addressed through the use of the community health worker model. CHWs that administered the survey were trusted individuals hired from participants’ communities. In addition, the survey was administered in private settings and participants were assured of data confidentiality. This ensured a level of trust and understanding between the interviewer and interviewee [24, 25].

## Limitations

There are several limitations to these results. First, findings were among enrolled Building Bridges participants who may have been more inclined to report willingness to participate in donation because of the relationship they had developed with the program’s Community Health Worker. Second, we observed that the lack of Arabic and Central African responses may be more reflective of the Community Health Worker’s own discomfort in asking the questions to their community. CHWs reported to receive feedback on why the questions were asked. For some populations, research has a negative image based on prior experience with government discrimination and experimentation. Additionally, in some communities biospecimens such as hair and nails have been used in religious curses. Further research could be conducted in this area to explore these underlying reasons for lower willingness to participate in research. Thirdly, participants reside in the North Texas area and may not be representative of other immigrant populations. Refugees in Texas are more likely to become uninsured after losing Refugee Medical Assistance eight months after arrival due to the lack of Medicaid expansion under the Affordable Care Act. This may result in lack of access to primary care providers and health information about the benefits of participating in clinical research. Finally, it is possible that despite concordant language and explanation, some participants may not have understood the question given their educational background and exposure to Western medicine. Stool samples and blood work are common experiences in healthcare. Upon arrival in the US for refugees, stool samples are required by the public health system to check for parasites, and blood samples to check for communicable diseases. Though there was a simple definition provided, there may have been confusion on the definition of biospecimen samples for research purposes only.

## Conclusion

This study provides information on the willingness of an underrepresented diverse immigrant population to participate in biospecimen donation for research purposes. Lower participation in screening and preventive practices for immigrant populations limit the cancer treatment and prevention strategies for the generalized population and specialized options for immigrants. Overall, the participants were willing to donate biospecimens. An increased willingness was seen in older immigrants and in female immigrants, though more research needs to be done to see if willingness translates to true donation. The inclusion of community leaders increased access to immigrants as well as hiring community health workers to facilitate the questions may be important factors in willingness to donate. Using our framework of culturally and linguistic appropriate outreach and education by trusted community members may be the key to including multiethnic immigrant populations in biomedical research. Our results support intentional inclusion of multicultural populations into medical research in general, and the addition of biospecimen collection in future phases of this project.
